# Dietary acid load and cirrhosis-related mortality: a prospective cohort study

**DOI:** 10.1038/s41598-024-53882-8

**Published:** 2024-02-14

**Authors:** Fereshteh Pashayee-Khamene, Zeinab Heidari, Danial Fotros, Azita Hekmatdoost, Sara Karimi, Saleheh Ahmadzadeh, Mehdi Saberifiroozi, Behzad Hatami, Zahra Yari

**Affiliations:** 1 Student Research Committee, Ahvaz Jundishapur University of Medical Sciences, Ahvaz, Iran; 2 Clinical Nutrition and Dietetics Department, Faculty of Nutrition Sciences and Food Technology, National Nutrition and Food Technology Research Institute, Shahid Beheshti University of Medical Sciences, Tehran, Iran; 3 Liver and Pancreato-Biliary Disease Research Center, Digestive Disease Research Institute, Shariati Hospital, Tehran University of Medical Sciences, Tehran, Iran; 4 Gastroenterology and Liver Diseases Research Center, Research Institute for Gastroenterology and Liver Diseases, Shahid Beheshti University of Medical Sciences, Tehran, Iran; 5 Department of Nutrition Research, National Nutrition and Food Technology Research Institute and Faculty of Nutrition Sciences and Food Technology, Shahid Beheshti University of Medical Sciences, West Arghavan St. Farahzadi Blvd., Sharake Qods, Tehran, Iran

**Keywords:** Cirrhosis, Mortality, Dietary acid load, PRAL, NEAP, Diseases, Gastroenterology, Medical research

## Abstract

As a global health concern, cirrhosis contributes significantly to morbidity and mortality. This prospective cohort study aimed to investigate the association between dietary acid load (DAL) and cirrhosis-related mortality. Present study was conducted on 121 patients with newly diagnosed cirrhosis who were followed up for 48 months. Anthropometric measures, nutritional status and dietary intakes were assessed and DAL was estimated based on potential renal acid load (PRAL) and net endogenous acid production (NEAP) scores. Crude and multivariable-adjusted hazard ratios (HR) with 95% confidence intervals (CI) were estimated using Cox proportional hazard analyses. Participants in the high PRAL and NEAP scores had significantly higher intakes of grains and lower intakes of fruits and vegetables. Also, the intake of dairy products and legumes, nuts and seeds decreased significantly with increasing NEAP score. After adjustment of all the confounders, the risk of mortality in the second and third tertiles of PRAL was 5.9 times and 10.97 higher than those in the first tertile, respectively (*P* trend: 0.006). Similarly, comparing the risk of mortality in the second and third tertiles with the first tertile of NEAP showed a 4.46-fold and 12.3-fold increased risk, respectively (*P* trend: 0.010). Our findings suggested that DAL was significantly associated with cirrhosis-related mortality and highlight the need for further research to understand the underlying mechanisms and establish optimal DAL levels in cirrhotic patients.

## Introduction

Cirrhosis is a chronic and progressive liver disease that affects millions of people worldwide and causes substantial morbidity and mortality^1^. Cirrhosis can lead to serious complications, such as portal hypertension, variceal bleeding, ascites, hepatic encephalopathy, and hepatocellular carcinoma, that often require hospitalization and increase the risk of mortality^2^. According to the Global Burden of Disease (GBD) Study in 2019, cirrhosis was responsible for 1.47 million deaths worldwide in 2019, an increase of 9.7% compared to 2017^3,4^. Likewise, based on the latest available statistics, in Iran, cirrhosis accounted for 1.42% of total deaths in 2017^5^. Therefore, it is important to identify the risk factors and preventive strategies for cirrhosis and its complications. Diet plays an important role in the etiology and management of liver cirrhosis, and it can be considered a low-cost and low-risk treatment plan to be applied to the majority of patients^6–10^.

Diet can contain acidic or alkaline load. Indeed, the idea that diet-induced acidosis can cause chronic disease has been a topic of interest for years^11–14^. Some observational studies have investigated the possible link between diets that produce acid with an increased risk of hypertension (HTN)^15,16^, osteoporosis^17,18^, insulin resistance (IR), and diabetes^19,20^. Moreover, a recent comprehensive study revealed a significant link between metabolic syndrome and dietary acid load (DAL)^21^. Nonetheless, the effects of DAL on cirrhosis, especially cirrhosis-related mortality, have not been well elucidated. DAL refers to the balance between acid-forming and alkaline-forming components in the diet, which can affect the acid–base balance in the body and potentially affect health^22^. DAL can be estimated based on two indices: net endogenous acid production (NEAP) and potential renal acid load (PRAL) based on dietary intake of five nutrients (protein, potassium, phosphorous, calcium, and magnesium)^23^. In this concept, animal foods such as meat, fish, and cheese, which are rich in acid-forming amino acids, increase the acid load of the diet, whereas most fruits and vegetables buffer the acid load of the diet due to their potassium content^13,22^.

It has been shown that diets with a high acid load can cause low-grade metabolic acidosis, which is associated with metabolic abnormalities^22^. These metabolic abnormalities, especially insulin resistance (IR)^24^, dysglycemia^19^, high blood pressure (HTN)^25^ and metabolic syndrome^21^, are considered risk factors for liver diseases such as cirrhosis. On the other hand, high consumption of red meat and low consumption of fruits and vegetables are characteristics of a diet with a high acid load, each of which alone can contribute to the development and progression of cirrhosis^26,27^.

In the current study, we aimed to investigate the possible association between DAL (based on PRAL and NEAP) and cirrhosis-related mortality using data from a prospective cohort study in cirrhotic patients.

## Methods and materials

### Study design and population

166 newly diagnosed cases (identified within 6 months of diagnose), aged over 18 years, willing to participate in the study were included in the present cohort study. Patients were recruited from two educational hospitals in Tehran, Iran. Exclusion criteria were: breastfeeding or pregnancy in women, history of renal failure, various types of cancer, diabetes mellitus, infectious diseases, cardiac disease, acquired immune deficiency syndrome, pancreatic insufficiency.

Patients were enrolled between 2016 and 2018 and followed up for 48 months until April 30, 2022. In order to track the occurrence of death or any other medical event, telephone calls were made to the participants annually. 45 patients were excluded for reasons including cancer diagnosis in the first year, missing or incomplete baseline data, high or low energy intake (< 500 or > 5000 kcal/day), and extreme body mass index (BMI) (< 15 or > 50 kg/m^2^). Finally, 121 patients (38 women and 83 men) were included in the final analysis.

The study protocol received approval from the National Nutrition and Food Technology Research Institute (NNFTRI) ethics committee (Ir.sbmu.nnftri.1396.186.) in accordance with the Declaration of Helsinki. Written informed consents were obtained from all participants, after providing explanations about the study protocol.

### Dietary assessment and dietary acid load calculation

The usual dietary intakes of the participants were collected through a face-to-face interview, using a reliable and valid food frequency questionnaire (FFQ) consisting of 168 items^28^. The frequency of consumption of each food during the last year was recorded based on the daily, weekly monthly and consumption of each food and converted into grams based on household measurements. Dietary data were assessed and analyzed by a skilled nutritionist using Nutritionist IV software. The average daily intake of energy and nutrients was calculated using The United States Department of Agriculture (USDA) food composition table (FCT).

The dietary acid load was estimated by the method previously developed based on two measures (PRAL and NEAP), using the following algorithms:

PRAL (mEq/d) = 0.4888 × dietary protein (g/d) + 0.0366 × dietary phosphorus (mg/d) − 0.0205 × dietary potassium (mg/d) − 0.0125 × calcium (mg/d) − 0.0263 × magnesium (mg/d)^29^.

Negative values indicate an alkaline-forming potential, while positive values reflect an acid-forming potential.

NEAP (mEq/d) = 54.5 × protein intake (g/d)/potassium intake (mEq/d) − 10.2^30^.

### Anthropometric, disease and nutritional status assessment

Baseline data including age, sex, alcohol and tobacco use, and the etiology of cirrhosis were collected at the beginning of the study. Weight was measured to the nearest 100g using digital scales and height was measured to the nearest 0.5 cm using a tape meter, while the subjects were minimally clothed, in a standing position without shoes. Body mass index (BMI) was calculated as weight (kg) divided by square of the height (m^2^).

The subjective global assessment (SGA) score was determined according to Destky et al.^31^ study, based on which the participants were categorized into three groups: well-nourished (A), moderately malnourished (B), and severely malnourished (C). Using clinical and biochemical parameters and based on Child–Pugh and model for end-stage liver disease (MELD) scores, the severity and prognosis of liver cirrhosis were evaluated^32^.

### Statistical analysis

Statistical analyses were performed using SPSS (SPSS Inc., Chicago, Illinois), *P* values < 0.05 were considered statistically significant. Means ± standard deviation for continuous variables and number (percentages) for categorical variables were compared using one-way analysis of variance (ANOVA) method and the chi-square test (χ2), respectively. Energy-adjusted PRAL and NEAP (score × 1000/energy intake) were applied in statistical analysis and were assigned as tertiles. Crude and multivariable-adjusted hazard ratios (HR) with 95% confidence intervals (CI) were estimated using Cox proportional hazard analyses. There models were applied to address potential confounders: Model 1: adjusted for sex (male, female) and age (continuous); Model 2: additionally adjusted for BMI (continuous), alcohol use (yes > 30 g/day, no < 30 g/day), and smoking (yes, no); and Model 3: further adjusted for Child–Pugh (A, B & C), MELD (continuous), and etiology (virus, autoimmune, other).

### Ethics approval and consent to participate

National nutrition and Food Technology Research Institute (NNFTRI) ethics committee approved the study protocol (Ir.sbmu.nnftri.1396.186.) in accordance with the Declaration of Helsinki. All participants provided written informed consent and were informed about the study.

## Results

General characteristics of study participants across the tertiles of PRAL and NEAP are provided in Table 1. During 3955 person-month of follow-up, we documented 43 deaths (7 women, 36 men). Liver failure was responsible for 47% of deaths, cardiovascular diseases 40%, carcinoma 3% and other causes for 10% of deaths. Regarding the etiology, 56% of participants' cirrhosis was attributed to the virus, 31% to autoimmunity, and 13% to other causes. Patients were more likely to be male (68.6%), although there was no significant difference between the PRAL and NEAP tertiles. The number of smokers and alcohol drinkers represent no significant differences. The average age and anthropometric parameters of the participants did not show any significant difference across tertiles of DAL. The severity of cirrhosis, according to MELD and Child, showed a significant difference between tertiles, except for Child in PRAL tertiles. Also, there was no significant difference in the severity of malnutrition based on SGA.

**Table 1:** Baseline General Characteristics of Study Participants by Tertile of DALs.

	PRAL (mEq/day)				NEAP (mEq/day)			
	Tertile 1 (n = 41)	Tertile 2 (n = 40)	Tertile 3 (n = 40)	P value	Tertile 1 (n = 177)	Tertile 2 (n = 177)	Tertile 3 (n = 177)	*P* value
Men, n (%)	27 (66)	26 (65)	30 (75)	0.564	28 (68)	29 (32)	26 (65)	0.769
Age (y)	55 ± 9.8	56.7 ± 13.4	52.7 ± 12.1	0.331	56.3 ± 9.9	54.3 ± 12.4	53.7 ± 13.2	0.578
Alcohol drinker	11 (27)	7 (19)	9 (24)	0.674	12 (30)	6 (16)	9 (24)	0.331
Smoker, %	16 (40)	14 (38)	17 (42)	0.916	18 (45)	13 (34)	16 (41)	0.618
MELD score	11.2 ± 4.2	9.5 ± 3	12.1 ± 4	0.026	10.75 ± 4.1	9.65 ± 2.8	12.2 ± 4	0.032
Child Pugh category (A/B/C)								
A	22 (69)	25 (78)	21 (60)	0.279	23 (68)	25 (86)	20 (55)	0.030
B, C	10 (31)	7 (22)	14 (40)		11 (32)	4 (14)	16 (44)	
Weight, kg	73.3 ± 13.6	71.7 ± 15.8	76.6 ± 19.4	0.406	75.6 ± 13.3	75.15 ± 18.7	70.8 ± 16.79	0.362
Height, cm	164.6 ± 8.6	164.6 ± 8.3	166.8 ± 7.9	0.397	165.4 ± 7.9	165 ± 9.1	165.6 ± 8.3	0.956
BMI, kg/m^2^	27.3 ± 4.9	26.6 ± 5.3	27.5 ± 5.8	0.725	27.9 ± 5.1	27.6 ± 5.6	25.9 ± 5.1	0.188
Subjective global assessment								
A	13 (32)	13 (33)	13 (33)	0.998	13 (32)	16 (40)	10 (25)	0.483
B	22 (54)	22 (55)	21 (52)		23 (56)	17 (42)	25 (62)	
C	6 (15)	5 (12)	6 (15)		5 (12)	7 (17)	5 (12)	

The results are described as mean ± standard deviation (ANOVA test) or number (%) (Chi-square test).

*PRAL* potential renal acid load, *NEAP* net endogenous acid production, *BMI* body mass7 index, *MELD* Model for end-stage liver disease.

In Table 2 dietary intakes of participants are compared across the tertiles of PRAL and NEAP. The intake of energy and macronutrients showed no significant difference across the tertiles of PRAL and NEAP, except for dietary protein in PRAL. Intakes of micronutrients including potassium, phosphorus, calcium, and magnesium were significantly different across the tertiles of NEAP (*P* < 0.05), except for phosphorus (*P* = 0.054). While, except for phosphorus (*P* = 0.014), the intake of other micronutrients was not significantly different between the PRAL tertiles.

**Table 2:** Dietary intakes of patients across tertiles of dietary acid–base load.

	PRAL (mEq/day)				NEAP (mEq/day)			
	Tertile 1 (n = 41)	Tertile 2 (n = 40)	Tertile 3 (n = 40)	P value	Tertile 1 (n = 41)	Tertile 2 (n = 40)	Tertile 3 (n = 40)	*P* value
Calorie (Kcal/d)	2435 ± 949	2228 ± 1303	2832 ± 1079	0.053	2502 ± 937	2654 ± 1342	2337 ± 1107	0.464
Carbohydrate (g/d)	393 ± 167	341 ± 203	418 ± 155	0.154	406 ± 161	404 ± 205	343 ± 161	0.195
Protein (g/d)	81 ± 34	80 ± 45	111 ± 45.6	0.001	82 ± 33	101 ± 51	90 ± 46	0.163
Fat (g/d)	71 ± 32	68 ± 46	87 ± 45	0.096	73.6 ± 35	80 ± 48	75 ± 42	0.705
Phosphorous (mg/d)	1501 ± 666	1514 ± 812	1934 ± 740	0.014	1579 ± 657	1880 ± 903	1488 ± 665	0.054
Potassium (mg/d)	4429 ± 1864	3595 ± 2177	4041 ± 1758	0.158	4537 ± 179	4491 ± 2250	3035 ± 1398	< 0001
Calcium (mg/d)	1058 ± 497	416 ± 251	784 ± 184	0.064	1166 ± 500	1306 ± 644	997 ± 494	0.046
Magnesium (mg/d)	460 ± 214	416 ± 251	484 ± 184	0.365	468 ± 208	515 ± 257	376 ± 161	0.014
Food groups								
Grains (g/d)	422 ± 226	419 ± 218	539 ± 194	0.019	420 ± 218	496 ± 230	464 ± 207	0.302
Fruits (g/d)	730 ± 375	553 ± 428	483 ± 298	0.011	743 ± 352	640 ± 422	379 ± 270	< 0.001
Vegetables (g/d)	409 ± 316	223 ± 156	266 ± 212	0.002	386 ± 319	313 ± 208	200 ± 158	0.003
Red meat (g/d)	15 ± 22	12 ± 18	25 ± 56	0.266	15 ± 21	18 ± 21	20 ± 56	0.821
Dairy products (g/d)	292 ± 243	387 ± 279	438 ± 340	0.075	364 ± 280	463 ± 324	288 ± 254	0.026
Legumes and nuts (g/d)	113 ± 81	86 ± 72	100 ± 72	0.290	109 ± 70	125 ± 87	66 ± 56	0.001
Protein to potassium ratio	0.014 ± 0.002	0.018 ± 0.004	0.019 ± 0.004	< 0.001	0.014 ± 0.003	0.017 ± 0.002	0.019 ± 0.005	< 0.001
Animal protein to potassium ratio	0.004 ± 0.002	0.006 ± 0.003	0.006 ± 0.003	0.001	0.005 ± 0.003	0.006 ± 0.002	0.006 ± 0.003	0.119
Plant protein to potassium ratio	0.098 ± 0.003	0.012 ± 0.004	0.012 ± 0.005	0.021	0.009 ± 0.003	0.01 ± 0.003	0.013 ± 0.005	< 0.001

The results are described as mean ± standard deviation using ANOVA test.

*PRAL* potential renal acid load, *NEAP* net endogenous acid production.

The comparison of intake of food groups also indicated significant differences in intake of grains, fruits, and vegetables among PRAL tertiles. There was an also significant difference in intake of food groups between the NEAP tertiles, except for grains and red meats. The ratio of total, animal and vegetable protein to potassium, showed a significant increase among both NEAP and PRAL tertiles, except for animal protein in NEAP tertiles.

Table 3 indicates multivariable-adjusted hazard ratios and 95% confidence intervals for cirrhosis-related mortality across tertiles of DAL. The number of deaths was substantially significantly increasing throughout the DALs tertiles. In model 1, after adjusting the results for age and sex, the risk of mortality increased significantly (*P* trend = 0.001) in those who were in the second (OR 6.6; 95% CI 0.8, 57.2) and third (OR 17.38; 95% CI 2.3, 130.3) tertiles of PRAL and NEAP (OR 4.7; 95% CI 1, 21.7 and OR 8.56; 95% CI 1.9, 38.8, respectively). Similar results were achieved in models 2 and 3. So that after adjustment of all confounders, comparing the risk of mortality in the second and third tertiles with the first tertile of PRAL and NEAP showed an increased risk.

**Table 3:** Hazard ratios for total mortality in each tertile of DAL.

PRAL	Tertiles of dietary acid load			*P* trend
	T1 (< 1.78)	T2 (1.78–16.15)	T3 (16.15 ≤)	
No. of deaths	9	14	20	0.032
Model 1	Ref	6.6 (0.8–57.2)	17.38 (2.3–130.3)	0.001
Model 2	Ref	7.5 (0.9–64.4)	16.6 (2.2–126)	< 0.001
Model 3	Ref	5.9 (0.6–56.9)	10.97 (1.4–84.8)	0.006

NEAP	T1 (< 34.38)	T2 (34.38–41.9)	T3 (41.9 ≤)	
No. of deaths	8	16	19	0.016
Model 1	ref	4.7 (1–21.7)	8.56 (1.9–38.8)	0.002
Model 2	ref	8.9 (1.1–74.6)	15.6 (1.9–125.2)	0.003
Model 3	ref	4.46 (0.45–44.1)	12.3 (1.38–108.4)	0.010

Cox proportional hazards regression models for estimating HRs and 95% CIs.

Model 1: adjusted for age and sex.

Model 2: additionally adjusted for BMI, smoking, alcohol.

Model 3: additionally adjusted for etiology, MELD and child.

## Discussion

In the present study for the first time, we showed that higher DAL (based on PRAL and NEAP) was associated with a higher mortality risk in patients with cirrhosis. In comparing the highest and lowest tertiles of NEAP and PRAL scores in a fully adjusted model, we found that being in the highest tertiles of NEAP or PRAL was associated with a significant increase in mortality risk. Although there is no literature regarding DAL and cirrhosis-related mortality, several studies have investigated the association between DAL and disease-specific mortality, like cardiovascular diseases (CVDs) and cancer^33–37^.

According to a study by Hejazi et al.^36^ on a large population-based cohort study, being in the highest and lowest DAL scores was significantly associated with increased total and CVD-caused mortality. Similarly, Xu et al.^37^ found that both excess acid and alkali load in the diet may be associated with an increased mortality risk, especially CVD-caused mortality, among Swedish adults (a U-shaped association). Furthermore, the recent study conducted by Fereidouni et al.^34^ indicated that an increase in dietary acid load may lead to an increased risk of CVD-related mortality. Aside from that, several studies have suggested that DAL may be associated with non-alcoholic fatty liver disease (NAFLD) progression^38–40^. However, this association was not significant in all studies, and one study reported that NEAP and PRAL were not associated with advanced fibrosis^39^.

The exact mechanisms by which DAL may influence cirrhosis and its complications are currently unclear. However, several factors may contribute to this association, such as insulin resistance (IR)^24^, hypertension (HTN)^25^, high consumption of red meat^26^, and low consumption of fruits and vegetables^27^. It has been demonstrated that high acid-load diets can cause chronic low-grade metabolic acidosis (MA), which is associated with metabolic abnormalities^22^. One of the consequences of low-grade MA is increased cortisol secretion^41^. Hypercortisolism increases the risk of various metabolic disorders, such as sarcopenia^42^, IR^43^, HTN^44,45^, and CVDs^46^.

Hepatic steatosis and IR are closely related, as IR is both a cause and a consequence of NAFLD^47,48^. In fact, IR increases adipose tissue lipolysis, resulting in an elevated influx of free fatty acids (FFAs) to the liver^49–51^. FFAs and other lipid intermediates, such as diacylglycerol (DAG) and ceramides, can induce lipotoxicity in hepatocytes^52,53^. To counteract this, hepatocytes convert FFAs into triglycerides and store them in the liver. As a consequence of this protective mechanism, plasma levels of FFAs are reduced, and lipotoxicity related to FFAs is prohibited^53,54^. However, when the hepatocytes are overwhelmed by the lipotoxic effects of other lipid intermediates, such as ceramides and DAG, they trigger inflammation, necrosis, and fibrosis in the liver^52^. Specifically, this process leads to the progression of NAFLD to non-alcoholic steatohepatitis (NASH), characterized by inflammation and fibrogenesis mediated by hepatic stellate cells^48,55^. HTN is another possible mechanism that links DAL and NAFLD. The results of the study conducted by Fou et al.^25^ showed that HTN was associated with higher rates of liver steatosis and fibrosis. Similarly, animal models also revealed that HTN might affect the progression of NASH^56^ and fibrosis^57^. Moreover, clinical evidence has suggested that HTN may contribute to the onset of NAFLD and the advancement of liver fibrosis^58,59^.

In addition, reducing red meat consumption may slow the progression of NAFLD and fibrosis, as continued red meat consumption was linked to a higher risk of hepatic fibrosis^60^. Additionally, the study by Daftari et al.^26^ demonstrated that lower intakes of animal protein are associated with lower mortality risks in patients with cirrhosis. On the other hand, consuming potassium-rich foods, such as fruits and vegetables, may favor muscle preservation, while acidosis may impair protein synthesis, increase proteolysis, and enhance amino acid oxidation, leading to a greater loss of muscle mass^61^. The relation between DAL and skeletal muscle mass has also been observed previously^62^. Preserving skeletal muscle could improve survival in patients with cirrhosis^63,64^. Finally, it is important to note that 40% of the deaths in this study were due to CVD, which has previously been shown to be associated with DAL, and some similar mechanisms have been proposed to explain its association^34,36,37^.

To the best of our knowledge, this is the first prospective cohort study to examine the relationship between DAL and mortality in patients with cirrhosis. The 4-year follow-up duration and the adjustment of several possible confounding factors are among the strengths of the study. However, some limitations should be considered. First, the small sample size limited the precision of the effect estimates. Therefore, the findings should be confirmed by larger studies and interpreted cautiously. Second, the use of the FFQ may be subject to recall bias and measurement errors in dietary intake assessment. Third, about 15% of the enrolled patients were lost to follow-up. Finally, as with most observational studies, residual and unmeasured confounding may affect the results. Thus, clinical trials are needed to provide more robust evidence on the differences in complications and mortality between different levels of DAL in these patients.

## Conclusion

In conclusion, our study provides valuable insights into the relationship between DAL and cirrhosis-related mortality, emphasizing the need for continued research to guide clinical interventions and preventive strategies. It was found that high DAL (based on PRAL and NEAP) is significantly associated with cirrhosis-related mortality. Further studies are warranted to elucidate the underlying mechanism(s) and optimal level of DAL in these patients.

## Data Availability

The datasets analyzed in the current study are available from the corresponding author on reasonable request.
